# Supplementary material for: Enhanced figure of merit in nanostructured (Bi,Sb)2Te3 with optimized composition, prepared by a straightforward arc-melting procedure
Source: Sci Rep. 2017 Jul 24;7:6277. doi: 10.1038/s41598-017-05428-4 (PMC5524901; doi:10.1038/s41598-017-05428-4)
Supplement: Supplementary file 1 — Supporting Information [file 41598_2017_5428_MOESM1_ESM.pdf]

SUPPORTING INFORMATION

Enhanced figure of merit in nanostructured $(Bi,Sb)_2Te_3$ with optimized composition, prepared by a straightforward arc-melting procedure

F. Serrano-Sánchez[1], M. Gharsallah[2], N.M. Nemes[3*], N. Biskup[3], M. Varela[3,4], J.L. Martínez[1], M.T. Fernández-Díaz[5], J.A. Alonso[1]

[1]*Instituto de Ciencia de Materiales de Madrid, C.S.I.C., Cantoblanco, E-28049 Madrid, Spain.*

[2]*Sfax University, National School of Engineers, B. P. W 3038, Sfax, Tunisia.*

[3]*Departamento de Física de Materiales, Universidad Complutense de Madrid, E-28040 Madrid, Spain.*

[4]*Instituto Pluridisciplinar & Instituto de Magnetismo Aplicado, Universidad Complutense de Madrid, E-28040 Madrid, Spain.*

[5] *Institut Laue Langevin, BP 156X, F-38042, Grenoble, France*

Table S1. Structural parameters for $BiSbTe_3$ refined in the R-3m space group (hexagonal setting) from NPD data collected at RT. Unit cell parameters: a = 4.3337(1) Å, c = 30.5072 (2) Å, V = 496.21 (4) Å^3, Z= 3. The discrepancy factors after the refinement are also included.

Fractional atomic coordinates and equivalent isotropic displacement parameters (Å^2)

	x	y	z	U_{eq}*	Occ. (<1)
Bi	0.00000	0.00000	0.4002(2)	0.021 (3)	0.67 (5)
Sb	0.00000	0.00000	0.4002 (2)	0.021 (3)	0.33 (5)
Te1	0.00000	0.00000	0.00000	0.012 (3)	0.50 (0)
Te2	0.00000	0.00000	0.7891 (1)	0.021 (4)	1.00 (0)

*Anisotropic displacement parameters (Å^2)

	U^{11}	U^{22}	U^{33}	U^{12}	U^{13}	U^{23}
Bi	0.018 (3)	0.018 (3)	0.028 (3)	0.009 (3)	0.00000	0.00000
Sb	0.018 (3)	0.018 (3)	0.028 (3)	−0.004 (3)	0.00000	0.00000
Te1	0.018 (4)	0.018 (4)	−0.001 (3)	0.009 (4)	0.00000	0.00000
Te2	0.026 (4)	0.026 (4)	0.011 (3)	0.013 (4)	0.00000	0.00000

Discrepancy factors

R_p= 5.71%, R_{wp}= 6.76%, R_{exp}=6.04%, R_{Bragg}=7.80%

Table S2. Structural parameters for $Bi_{0.5}Sb_{1.5}Te_3$ refined in the R-3m space group (hexagonal setting) from NPD data collected at RT. Unit cell parameters: a = 4.3008(1) Å, c = 30.5007 (2) Å, V = 488.59 (4) $Å^3$, Z= 3. The discrepancy factors after the refinement are also included.

Fractional atomic coordinates and equivalent isotropic displacement parameters ($Å^2$)

	x	*y*	*z*	U_{eq}*	Occ. (<1)
Bi	0.00000	0.00000	0.3997 (2)	0.025 (3)	0.44 (5)
Sb	0.00000	0.00000	0.3997 (2)	0.025 (3)	0.56 (5)
Te1	0.00000	0.00000	0.00000	0.011 (3)	0.50 (0)
Te2	0.00000	0.00000	0.7882 (1)	0.020 (3)	1.00 (0)

*Anisotropic displacement parameters ($Å^2$)

	U^{11}	U^{22}	U^{33}	U^{12}	U^{13}	U^{23}
Bi	0.020 (3)	0.020 (3)	0.035 (3)	0.010 (3)	0.00000	0.00000
Sb	0.020 (3)	0.020 (3)	0.035 (3)	−0.006 (3)	0.00000	0.00000
Te1	0.014 (4)	0.014 (4)	0.005 (3)	0.007 (4)	0.00000	0.00000
Te2	0.021 (3)	0.021 (3)	0.017 (3)	0.011 (3)	0.00000	0.00000

Discrepancy factors

R_p= 5.48%, R_{wp}= 6.92%, R_{exp}=6.41%, R_{Bragg}=6.94%

Table S3. Structural parameters for Sb_2Te_3 refined in the R-3m space group (hexagonal setting) from NPD data collected at RT. Unit cell parameters: a = 4.2673 (2) Å, c = 30.4510 (19) Å, V = 480.21 (4) Å^3, Z= 3. The discrepancy factors after the refinement are also included.

Fractional atomic coordinates and equivalent isotropic displacement parameters (Å^2)

	x	*y*	*z*	U_{eq}*	Occ. (<1)
Sb	0.00000	0.00000	0.3984 (3)	0.025 (3)	1.00 (0)
Te1	0.00000	0.00000	0.00000	0.014 (4)	0.50 (0)
Te2	0.00000	0.00000	0.7877 (2)	0.020 (3)	1.00 (0)

*Anisotropic displacement parameters (Å^2)

	U^{11}	U^{22}	U^{33}	U^{12}	U^{13}	U^{23}
Sb	0.021 (3)	0.021 (3)	0.033 (4)	0.010 (3)	0.00000	0.00000
Te1	0.018 (5)	0.018 (5)	0.008 (4)	0.009 (5)	0.00000	0.00000
Te2	0.020 (4)	0.020 (4)	0.022 (3)	0.010 (4)	0.00000	0.00000

Discrepancy factors

R_p= 5.20%, R_{wp}= 6.53%, R_{exp}=6.15%, R_{Bragg}=9.44%

Figure S1. Observed (crosses), calculated (full line), and difference (at the bottom) NPD profiles for $BiSbTe_3$ at RT

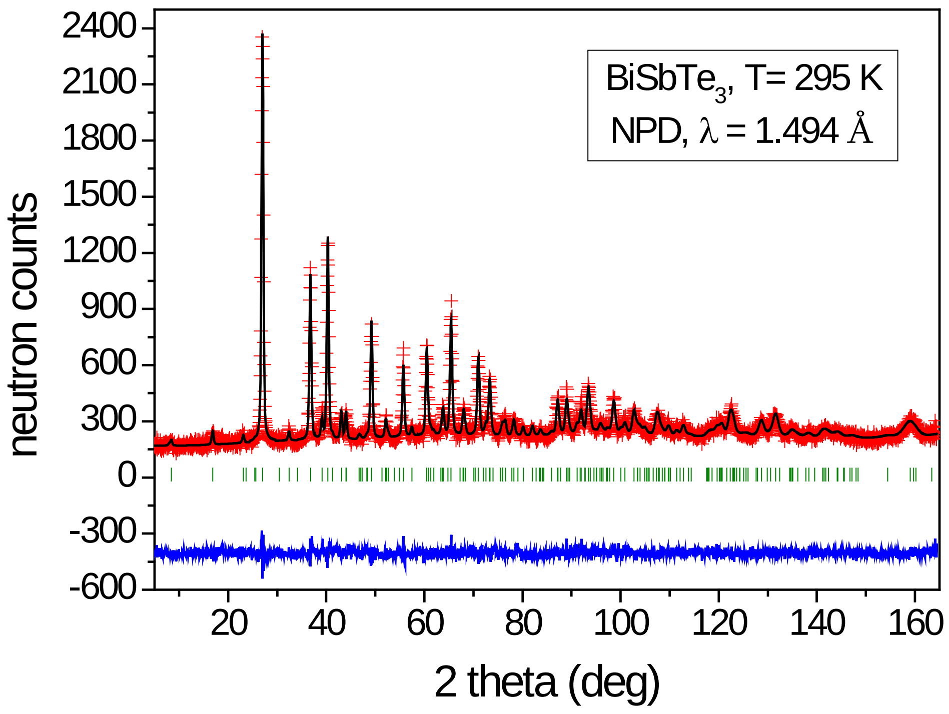

Fig. S2 Observed (crosses), calculated (full line), and difference (at the bottom) NPD profiles for Sb_2Te_3 at RT

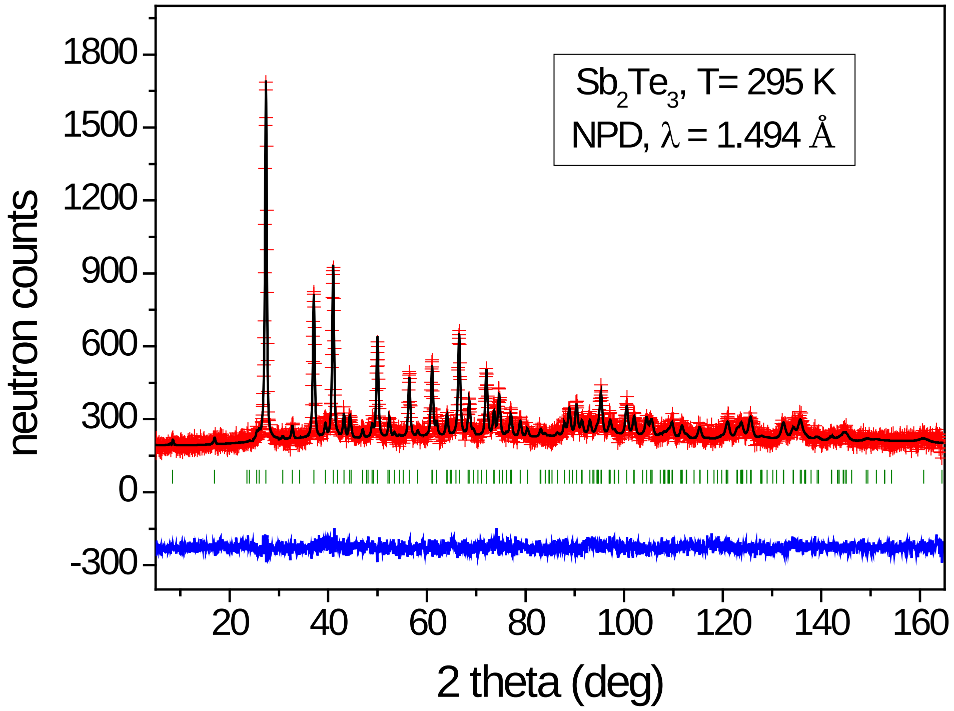

Fig. S3 Low resolution TEM image and corresponding diffraction pattern showing ring-type powder pattern, and EDX spectrum of the grain studied in more detail in Fig. 5 of the main article, with nominal composition of $Bi_{0.35}Sb_{1.65}Te_3$. The composition is found to be $Bi_{0.51}Sb_{1.52}Te_3$ in this grain. The composition of other grains (not shown) was also found to be consistent with $Bi_{0.5}Sb_{1.5}Te_3$.

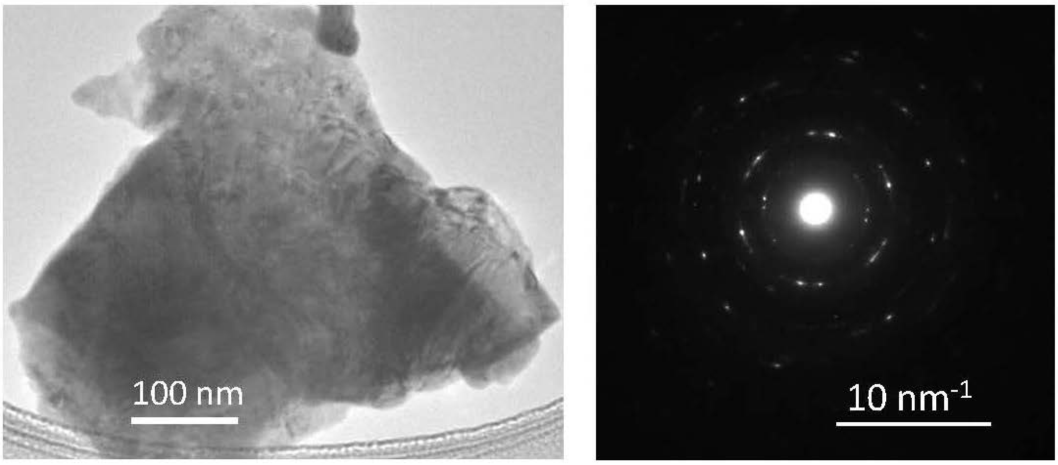

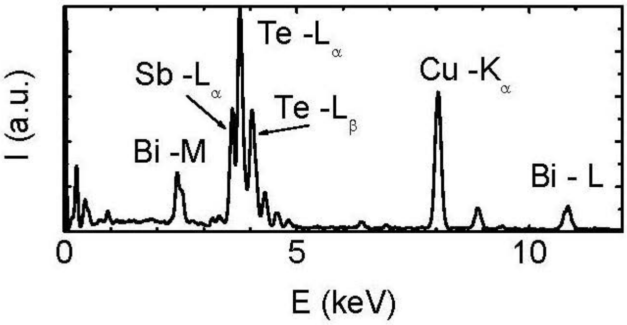

Element	Weight%	Atomic%
Sb L	27.51	30.30
Te L	56.65	59.53
Bi L	15.84	10.17
Totals	100.00	
